# LINC01087 indicates a poor prognosis of glioma patients with preoperative MRI

**DOI:** 10.1007/s10142-021-00812-w

**Published:** 2021-11-24

**Authors:** Wangsheng Chen, Fei Wang, Jianhua Zhang, Changqing Li, Lan Hong

**Affiliations:** 1 Department of Radiology, Hainan General Hospital, Affiliated Hainan Hospital of Hainan Medical College, No.19 Xiuhua Road, Xiuying District, Haikou, 570311 Hainan China; 2 Department of Radiology, Shanghai East Hospital, Tongji University School of Medicine, Shanghai, 200120 China; 3 Department of Gynecology, Hainan General Hospital, Affiliated Hainan Hospital of Hainan Medical College, No.19 Xiuhua Road, Xiuying District, Haikou, 570311 Hainan China

**Keywords:** Glioma, Long intergenic non-coding RNA 01,087, microRNA-1277-5p, Alkaline ceramidase 3, Prognosis, Biological function, Tumor growth

## Abstract

Long intergenic non-coding RNA 01,087 (LINC01087) has been concerned as an oncogene in breast cancer, while its mechanism in glioma has been little surveyed. Thus, we searched the prognostic value and functional action of LINC01087 in glioma. Glioma patients after preoperative MRI diagnosis were enrolled, and LINC01087, microRNA (miR)-1277-5p, and alkaline ceramidase 3 (ACER3) levels were tested in glioma cancer tissue. The correlation between LINC01087 expression and the survival of patients were analyzed. LINC01087, miR-1277-5p, and ACER3 levels in U251 cells were altered via transfection, and cell malignant phenotypes were monitored. The relationship between miR-1277-5p and LINC01087 or ACER3 was detected. The LINC01087 and ACER3 expression was in up-regulation and the miR-1277-5p expression was in down-regulation in clinical glioma samples. High expression of LINC01087 was associated with poor prognosis of glioma patients with preoperative MRI. LINC01087 silencing restrained tumor malignancy in glioma cells. Mechanistically, LINC01087 directly interacted with miR-1277-5p. ACER3 was a known target of miR-1277-5p. Moreover, rescue assays reveal that miR-1277-5p overexpression (or ACER3 overexpression) reversed the effects of LINC01087 upregulation (or miR-1277-5p upregulation) on glioma cells. LINC01087 has prognostic significance in glioma and silencing LINC01087 deters glioma development through elevating miR-1277-5p to reduce ACER3 expression.

## Introduction

Gliomas are intrinsic intracranial tumors derived from glial progenitor cells, of which glioblastoma is the most malignant form and of ill repute for treatment resistance (Gusyatiner and Hegi 2018). Genetic factors, ionizing radiation, and history of allergies are generally considered to be known risk factors (Davis 2018). The survival rates differ among all glioma subtypes, and glioblastoma has the worst overall survival, with extreme low 5-year survival rate after diagnosis (Ostrom et al. 2014). The three mainstays for the treatment of high-grade gliomas comprise of maximum surgical resection, external beam radiation therapy, and chemotherapy (Bush et al. 2017). Unexpectedly, complications associated with tumor treatment happen, such as progressive brain volume reduction, leukoencephalopathy, neurovascular complications, and secondary tumors (Tom et al. 2019). On that account, it is imperative to advance novel approaches for glioma.

LncRNA is a functional RNA molecule that participates in glioma, and lncRNA-regulated miRNA and signaling pathways offer a prospect of developing therapies for glioma (Dang et al. 2018). Xuan Wang et al. have proposed that targeted-depletion of LINC00473 deters glioma progression via interacting with miR-195-5p (Wang et al. 2020). Shi J et al. also announce that a cellular therapy based on LINC00174/miR-152-3p interaction could regulate glycolysis and tumorigenicity in glioma (Shi et al. 2019). Notably, Liu X et al. have illustrated the promoting effects of LINC00689 on metastasis and glycolysis of glioma cells by binding to miR-338-3p (2019). LINC01087 imposes the carcinogenic effect on breast cancer, which is negatively related to the patient’s prognosis (She et al. 2020), and could modify the biological functions of cells (Tripathi et al. 2020). According to a research related to gastric cancer, miR-1277-5p could rescue growth and metastasis of tumor cells (Wei et al. 2020). But, the interaction between LINC01087 and miR-1277-5p in glioma slides into obscurity. It has been tested that ceramidase inhibitor induces cytotoxicity and apoptosis of giloma cells (Kus et al. 2018). Alkaline ceramidase 3 (ACER3), a member of ceramidases family, is localized to the endoplasmic reticulum and the Golgi complex (Coant et al. 2017). ACER3 often expresses highly in the malignant progression of tumors, such as acute myeloid leukemia (Chen et al. 2016). Interestingly, ACER3 involves in tumorigenesis-related mechanism and modifies tumor progression and radiosensitivity through regulation by lncRNA and miRNA (Yang et al. 2020). For these reasons, we examined the prognostic value of LINC01087 in glioma and also explored whether LINC01087/miR-1277-5p/ACER3 axis could modulate the biological activities of glioma cells.

## Methods and materials

### Ethics statement

The project was approved by the Ethics Committee of Hainan General Hospital, and written informed consent was obtained from all patients.

### Clinical tissues

A total of 80 fresh glioma samples and adjacent normal tissues were collected in Hainan General Hospital from patients diagnosed by preoperative MRI, and maintained in liquid nitrogen (− 196 °C). Inclusion criteria were as follows: At least two experienced pathologists examined the patient’s tissue; the patient underwent radical resection with a clear surgical margin, and the distance between adjacent non-tumor tissue and the tumor edge was at least 1 cm; patients with available follow-up information; no patients had received anti-cancer treatment before surgery, and history of other cancers. Exclusion criteria were as follows: patients suffering from severe diseases or chronic diseases. The overall survival time was determined as the survival time after surgery.

### Cell culture and transfection

Glioma cell lines (U87, LN229, and U251) and human glial cell line HEB (China Center for Type Culture Collection, Wuhan, China) were cultured in Dulbecco’s modified Eagle medium (DMEM) containing 10% fetal bovine serum (FBS). The medium were supplied by Gibco & Thermo Fisher Scientific (CA, USA).

Glioma cells were transfected using Lipofectamine 3000 (Invitrogen, CA, USA). The oligonucleotides and plasmids used for transfection included small interfering RNA (si)-LINC01087, si-negative control (NC), miR-1277-5p mimic, mimic NC, short hairpin RNA (sh)-ACER3, sh-NC, overexpression (oe)-LINC01087 + mimic NC, oe-LINC01087 + miR-1277-5p mimic, miR-1277-5p mimic + oe-NC, or miR-1277-5p mimic + oe-ACER3. miR-1277-5p mimic and mimic NC were produced by RiboBio (Guangzhou, China); si-LINC01087, oe-LINC01087, and si-NC were designed by Genepharma (Shanghai, China); and oe-NC, oe-ACER3, sh-ACER3, and sh-NC were constructed by Sangon (Shanghai, China).

### Cell counting kit (CCK)-8 assay

CCK-8 reagent (Sigma-Aldrich, MO, USA) was employed for cell proliferation detection. After culture of 24, 48, 72, and 96 h, cells (2 × 10^3^ cells/well) were appended with CCK-8 solution (10 μL/well), and absorbance at 450 nm was recorded on a microplate reader (Zhang et al. 2020).

### Colony formation assay

Cells were cultivated for 12 days on a 6-well plate. After fixation with paraformaldehyde, cells were stained with crystal violet and the number of colonies was counted manually.

### Transwell assay

Cells were placed in serum-free DMEM for 12 h. The Transwell chamber was put in 24-well plates. The lower chamber was covered with 20% FBS-DMEM (500 µL), and meanwhile cells (1 × 10^5^, 100 µL) were placed in each well of the upper chamber (containing serum-free DMEM). After 48 h, migrated cells were fixed with 4% paraformaldehyde, followed by staining with 0.1% crystal violet. Finally, the cells in 5 fields of view were counted under an inverted microscope (Liu et al. 2020). Invasion assay was conducted with the upper chamber pre-coated with 10 µL Matrigel (Corning, NY, USA).

### Flow cytometry

Cells collected after trypsinization and centrifugation were resuspended in 1 × binding buffer, and detected according to the Annexin V-fluorescein isothiocyanate/propidium iodide kit (BD Pharmingen, CA, USA). Cell apoptosis was examined using a flow cytometer (BD Biosciences, NJ, USA).

### Reverse transcription quantitative polymerase chain reaction (RT-qPCR)

Total RNA collected by Trizol (Invitrogen) was transformed into cDNA. Reverse transcription of mRNA and miRNA was performed using PrimeScript RT Reagent Kit and Mir-X miRNA First-Strand Synthesis Kit (Takara; Dalian, China), respectively, according to the manufacturer’s protocol. Quantitative PCR was performed using SYBR Premix EX Taq Kit (Takara) on an ABI Prism 7900HT Real-Time System (Applied Biosystems Inc; Shanghai, China). Gene levels were processed using 2^−ΔΔCt^ method and standardized to U6 or glyceraldehyde-3-phosphate dehydrogenase (GAPDH). The primer sequences (RiboBio) are shown in Table 1.

**Table 1:** Primer sequences for RT-PCR analysis

Genes	Primers (**5**′–**3′**)
LINC01087	F: CAAAGAGCAACCAGCCA
	R: AACCAGTACCAGCCACTA
miR-1277-5p	F: AAATATATATATATATGTACGTAT
	R: universal primer
ACER3	F: CAATGTTCGGTGCAATTCAGAG
	R: GGATCCCATTCCTACCACTGTG
GAPDH	F: GCCATCACTGCCACCCAGAAGACTG
	R: CATGAGGTCCACCACCCTGTTGCTG
U6	F: CTCGCTTCGGCAGCACA
	R: AACGCTTCACGAATTTGCGT

*LINC01087*, long intergenic non-coding RNA 01,087; *miR-1277-5p*, microRNA-1277-5p; *ACER3*, alkaline ceramidase 3; *GAPDH*, glyceraldehyde-3-phosphate dehydrogenase

### Western blot assay

Cells or tissues were lysed with radio-immunoprecipitation assay lysis buffer (labscinece, Shanghai, China), and the protein contect was quntified using a BCA protein assay kit (Pierce, IL, USA). After separation by sodium dodecyl sulfate polyacrylamide gel electrophoresis, the protein sample was transferred to a polyvinylidene fluoride membrane, blocked, and reacted with anti-ACER3 (1:500, Saierbio, Beijing, China) or anti-GAPDH (1:1000, Abcam, MA, USA). After reaction with the secondary antibody (1:1000, Abcam), the protein level was detected by enhanced chemiluminescence (Millipore, MA, USA) (Yang et al. 2020).

### Dual luciferase reporter gene assay

LINC01087 wild-type (WT) and LINC01087 mutant (MUT), and ACER3 wild-type (WT) and ACER3 mutant (MUT) fragments containing miR-1277-5p binding sites were cloned into the pmirGLO vector (Promega, Madison, WI, USA) to construct reporter plasmids. Lipofectamine 3000 (Invitrogen) was applied to perform co-transfection of U251 cells with miR-1277-5p mimic/NC and LINC01087-WT/MUT or ACER3-WT/MUT, thus to calculate the luciferase activity (Li et al. 2020).

### RNA immunoprecipitation (RIP) assay

Using Magna RIP™ kit (Millipore), RIP detection was conducted with Ago2 antibody (Abcam) or immunoglobulin G antibody. Finally, the immunoprecipitated RNA was confirmed using RT-qPCR.

### Statistical analysis

Statistical analysis was performed using GraphPad Prism 8.3.0 (GraphPad Software, Inc.) and SPSS 22.0 (IBM, NY, USA). *t* test was performed to compare the differences between two groups, and analysis of variance and Tukey’s post hoc test were conducted to multiple data comparisons. The correlation between LINC01087 expression and patients’ clinicopathological characteristics was determined by chi-square test or Fisher's exact test while that between LINC01087 expression and patients’ overall survival by Kaplan–Meier. Pearson test was applied to correlation analysis. *P* < 0.05 meant statistical significance. The data were expressed as mean ± standard deviation.

## Results

### Increased LINC01087 in glioma patients is associated with poor prognosis

**Fig. 1:**
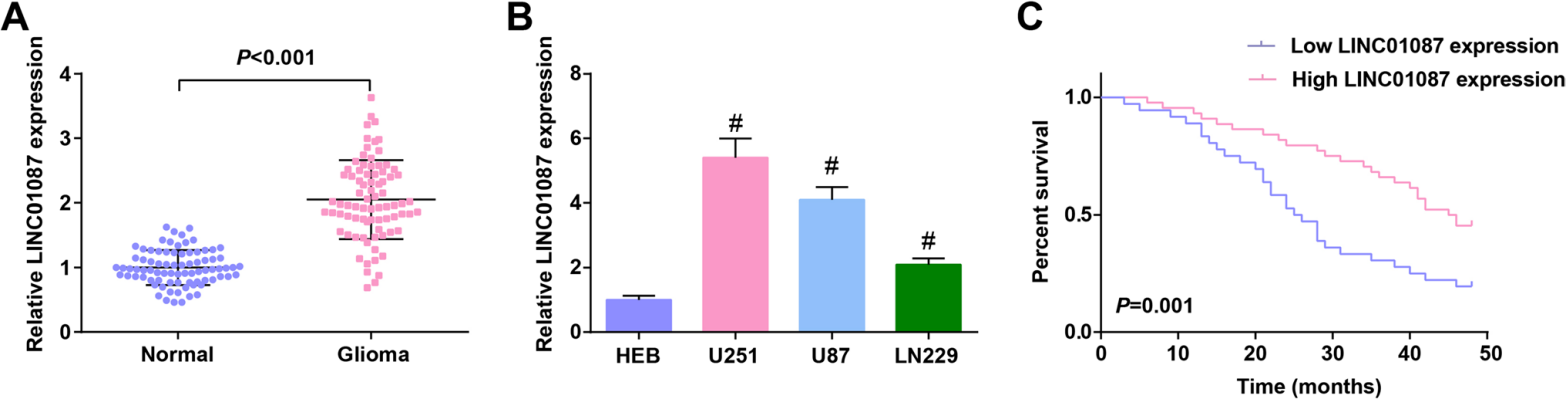
Increased LINC01087 in glioma patients is associated with poor prognosis. **A** LINC01087 expression level in glioma tissue; **B** LINC01087 expression level in glioma cell lines; **C** Kaplan-Meier survival curvese for glioma patients with high and low LINC01087 expression. The data were represented by mean ± standard deviation; #*P* < 0.05 vs. HEB cells

LINC01087 is highly expressed in breast cancer, indicating a poorer survival time of patients (She et al. 2020). We measured LINC01087 expression in 80 patients with glioma and found that LINC01087 was upregulated in glioma tissues (Fig. 1A). The same expression trend was also detected in glioma cell lines (U87, LN229, and U251) (Fig. 1B). U251 cell line expressing relative high LINC01087, therefore, it was selected for follow-up studies.

The glioma patients were divided into high expression group (*n* = 36) and low expression group (*n* = 44) according to the average LINC01087 expression level, and the correlation between LINC01087 expression and clinical parameters (Table 2) of glioma patients was determined. It was revealed that high expression of LINC01087 was related to tumor growth and WHO staging. Moreover, Kaplan–Meier analysis validated that the overall survival of glioma patients with high LINC01087 expression was poor (Fig. 1C).

**Table 2:** Correlation between LINC01087 expression and clinicopathological characteristics of patients with glioma

Parameters	Case	LINC01087		*P* value
		High expression (*n* = 36)	Low expression (*n* = 44)	
Age (years)				
< 45	43	24	19	0.822
≥ 45	37	22	15	
Gender				
Male	32	15	17	0.166
Female	48	31	17	
Tumor size				
≤ 5 cm	41	13	28	0.024
> 5 cm	39	23	16	
WHO stage				
I–II	49	15	34	0.001
III–IV	31	21	10	

Enumeration data were analyzed using chi-square test or  Fisher's exact test

### Reducing LINC01087 levels in glioma cells restrains tumor malignancy

**Fig. 2:**
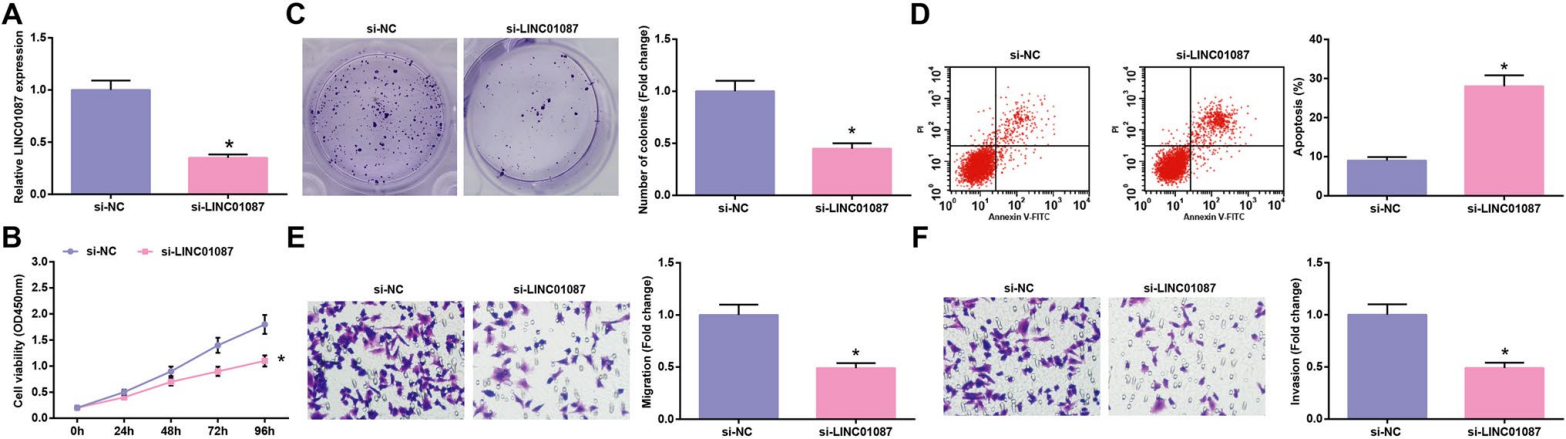
Reducing LINC01087 levels in glioma cells restrains tumor malignancy. **A** LINC01087 expression levels assessed by RT-qPCR; **B**–**C** Cell proliferation determined by CCK-8 and colony formation assays; **D** Cell apoptosis assessed by flow cytometry; **E**–**F** Cell migration and invasion assessed by Transwell assay. The data were represented by mean ± standard deviation; *P < 0.05 vs. the si-NC group

si-LINC01087 or si-NC was transfected into U251 cells, and RT-qPCR analysis confirmed that LINC01087 level was reduced by si-LINC01087 (Fig. 2A). CCK-8 and colony formation assay, along with flow cytometry and Transwell assay, were of utility to test cell proliferation, apoptosis, invasion, and migration. By knocking down LINC01087, U251 cells were characterized by inhibited proliferation (Fig. 2B, C), promoted apoptosis (Fig. 2D), and weakened migration and invasion ability (Fig. 2E, F). Shortly, reducing LINC01087 levels in glioma cells restrained tumor malignancy.

### LINC01087 could directly bind to miR-1277-5p

**Fig. 3:**
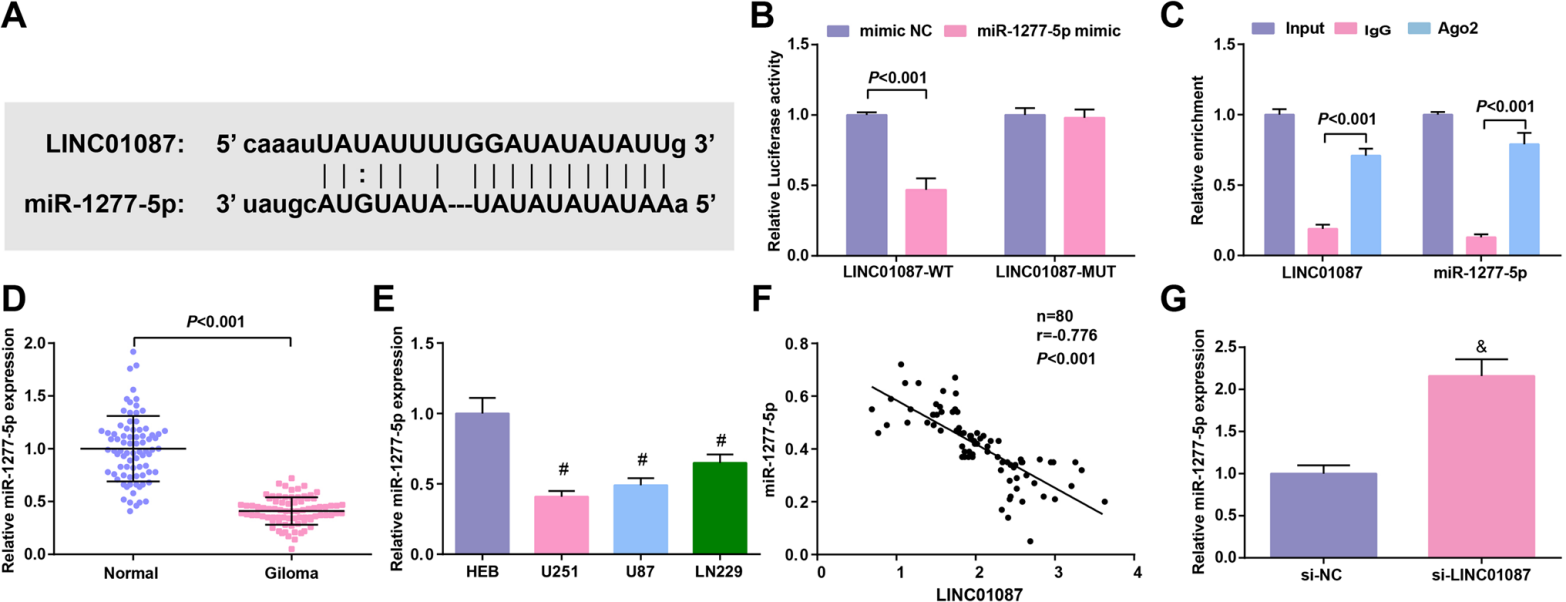
LINC01087 could directly bind to miR-1277-5p. **A** Binding sites between LINC01087 and miR-1277-5p predicted by Starbase; **B** The binding of LINC01087 and miR-1277-5p verified by dual luciferase reporter assay; **C** The binding of LINC01087 and miR-1277-5p verified by RNA pull-down experiment; **D** miR-1277-5p expression in glioma tissue assessed by RT-qPCR; **E** miR-1277-5p expression in glioma cell lines; **F** The correlation between the expression of LINC01087 and miR-1277-5p analyzed by Pearson test; **G** miR-1277-5p expression in U251 cells after silencing LINC01087. The data were represented by mean ± standard deviation; *P < 0.05 vs. the mimic NC group; #*P* < 0.05 vs. HEB cells; &*P* < 0.05 vs. the si-NC group

We found the binding site between LINC01087 and miR-1277-5p through the Starbase website (Fig. 3A). To verify this result, we performed dual luciferase reporter experiment and observed that miR-1277-5p-mimic caused an inhibition for the luciferase activity of LINC01087-WT (Fig. 3B). Also, RIP analyzed that both LINC01087 and miR-1277-5p could bind to Ago2 (Fig. 3C), validating the binding of LINC01087 to miR-1277-5p.

Then, miR-1277-5p levels in tissues and cells were evaluated, and it was examined to downregulate in glioma tissue and glioma cells (Fig. 3D, E). Based on Pearson test, we found a negative correlation between LINC01087 and miR-1277-5p expression in glioma tissues (Fig. 3F). With regard to the regulatory relationship between LINC01087 and miR-1277-5p, we adopted RT-qPCR to detect the expression change of miR-1277-5p after silencing LINC01087, and finally tested that reduced LINC01087 heightened miR-1277-5p expression (Fig. 3G).

### Elevating miR-1277-5p levels blunts the growth of glioma cells

**Fig. 4:**
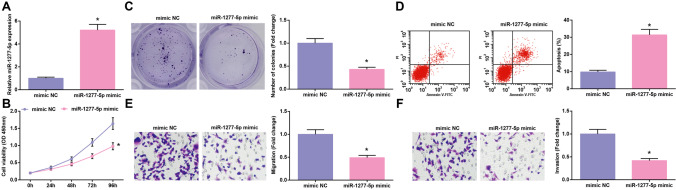
Elevating miR-1277-5p levels blunts the growth of glioma cells. **A** miR-1277-5p expression levels assessed by RT-qPCR; **B**–**C** Cell proliferation determined by CCK-8 and colony formation assays; **D** Cell apoptosis assessed by flow cytometry; **E**–**F** Cell migration and invasion assessed by Transwell assay. The data were represented by mean ± standard deviation; *P < 0.05 vs. the mimic NC group

Also, miR-1277-5p mimic or mimic NC was introduced into U251 cells to assess the mechanism of miR-1277-5p in glioma. RT-qPCR firsts confirmed the increase of miR-1277-5p expression in U251 cells induced by miR-1277-5p mimic (Fig. 4A). Next, cell experiments disclosed that U251 cells containing increased miR-1277-5p presented inhibited biological functions (Fig. 4B–F). To sum up, elevating miR-1277-5p levels blunted the growth of glioma cells.

### ACER3 is a target of miR-1277-5p

**Fig. 5:**
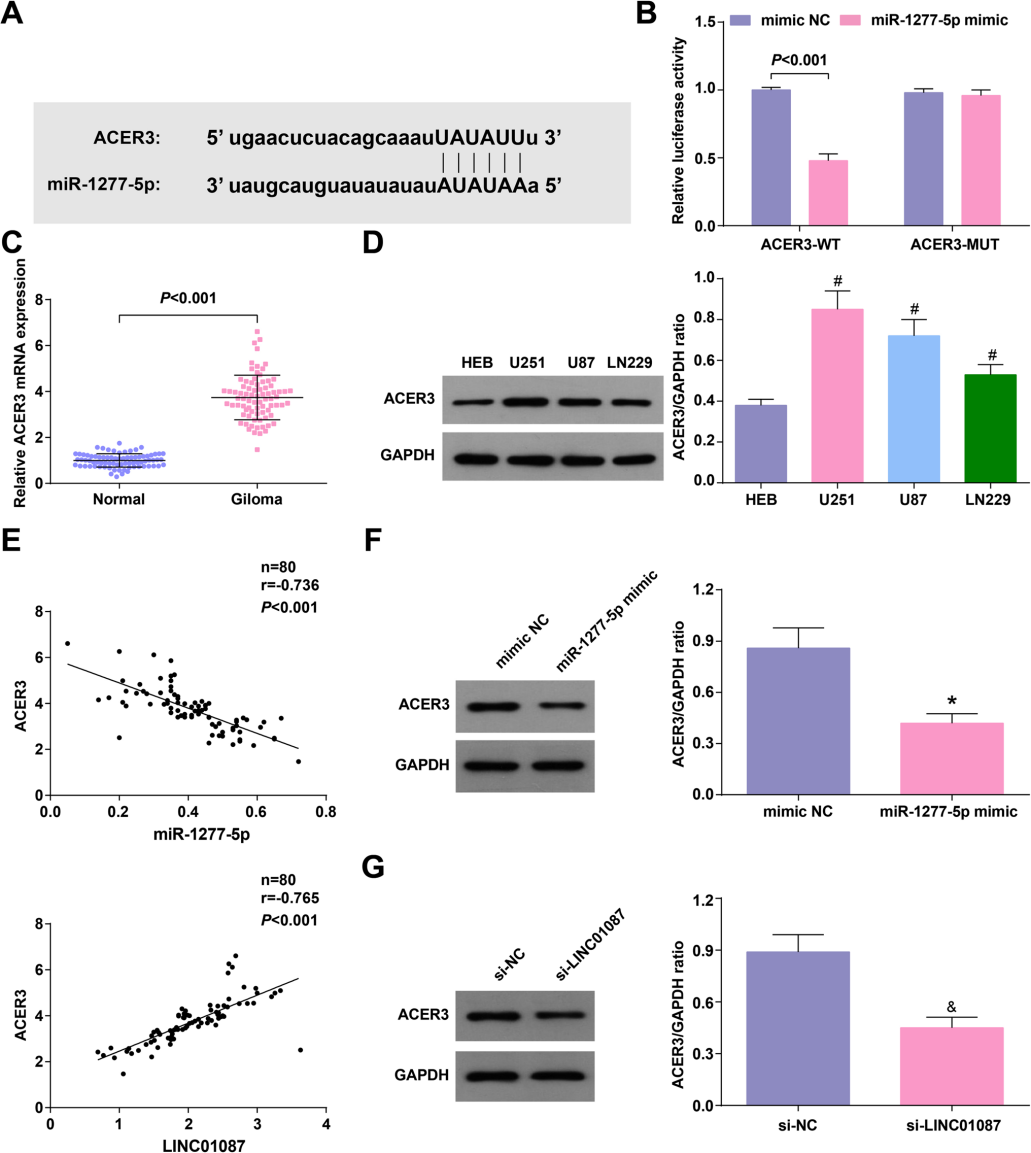
ACER3 is a target of miR-1277-5p. **A** The targeting site between miR-1277-5p and ACER3 analyzed by Starbase; **B** The binding of miR-1277-5p and ACER3 verified by dual luciferase reporter assay; **C** ACER3 mRNA expression level in glioma tissue and normal tissue assessed by RT-qPCR; **D** ACER3 mRNA and protein expression level in glioma cell lines and HEB assessed by RT-qPCR and Western blot; **E** The correlation between miR-1277-5p and ACER3, LINC01087 and ACER3 in glioma tissue analyzed by Pearson test; **F**–**G** ACER3 protein expression analyzed by Western blot; The data were represented by mean ± standard deviation; *P < 0.05 vs. the mimic NC group; #*P* < 0.05 vs. HEB cells; &*P* < 0.05 vs. the si-NC group

Through the Starbase website, we found that miR-1277-5p had binding site with ACER3 (Fig. 5A). In dual luciferase reporter assay, miR-1277-5p-mimic could reduce the luciferase activity of ACER3-WT (Fig. 5B). When evaluating ACER3 expression in glioma, it was identified that ACER3 was high-expressed in glioma tissues and cell lines (Fig. 5C, D). Pearson analysis declared that ACER3 expression was negatively correlated with miR-1277-5p expression, but positively correlated with LINC01087 expression in glioma tissue (Fig. 5E). In addition, transfection with miR-1277-5p-mimic or si-LINC01087 resulted in a decrease on ACER3 expression in U251 cells (Fig. 5F, G). Overall, ACER3 was regulated by miR-1277-5p and LINC01087.

### Silencing ACER3 limits the malignant phenotype of glioma cells

**Fig. 6:**
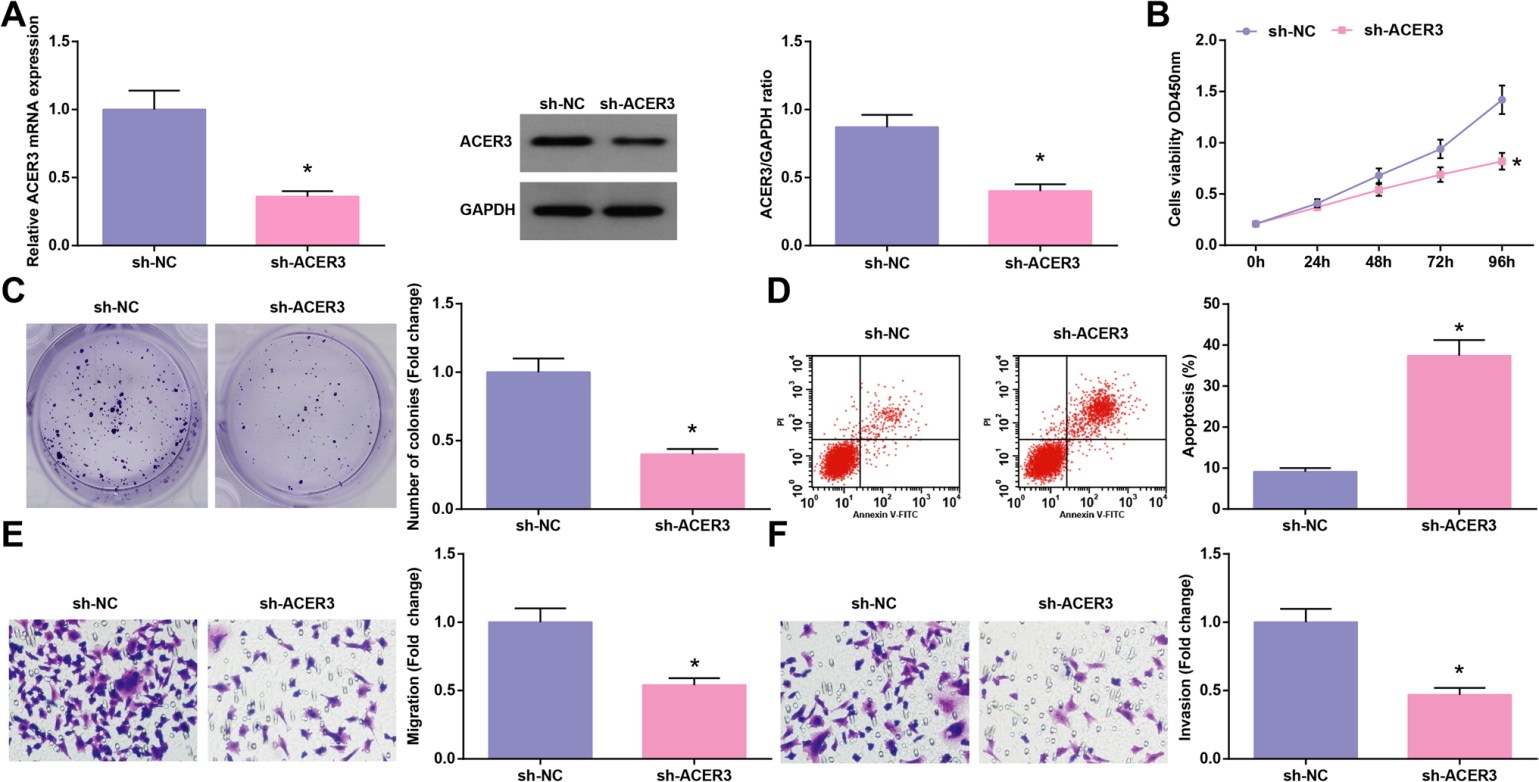
Silencing ACER3 limits the malignant phenotype of glioma cells. **A** ACER3 mRNA and protein expression levels assessed by RT-qPCR and Western blot; **B**–**C** Cell proliferation determined by CCK-8 and colony formation assays; **D** Cell apoptosis assessed by flow cytometry; **E**–**F** Cell migration and invasion assessed by Transwell assay. The data were represented by mean ± standard deviation; *P < 0.05 vs. the sh-NC group

U251 cells were transfected with sh-ACER3 or sh-NC, and ACER3 expression was successfully inhibited by sh-ACER3 (Fig. 6A). Then, experimental analysis displayed that after inhibition of ACER3 in U251 cells, cell proliferation, migration, and invasion were decelerated while apoptosis was accelerated (Fig. 6B–F). Briefly, silencing ACER3 limited the malignant phenotype of glioma cells.

### LINC01087-mediated tumorigenesis in glioma is mediated by miR-1277-5p-targeted ACER3

**Fig. 7:**
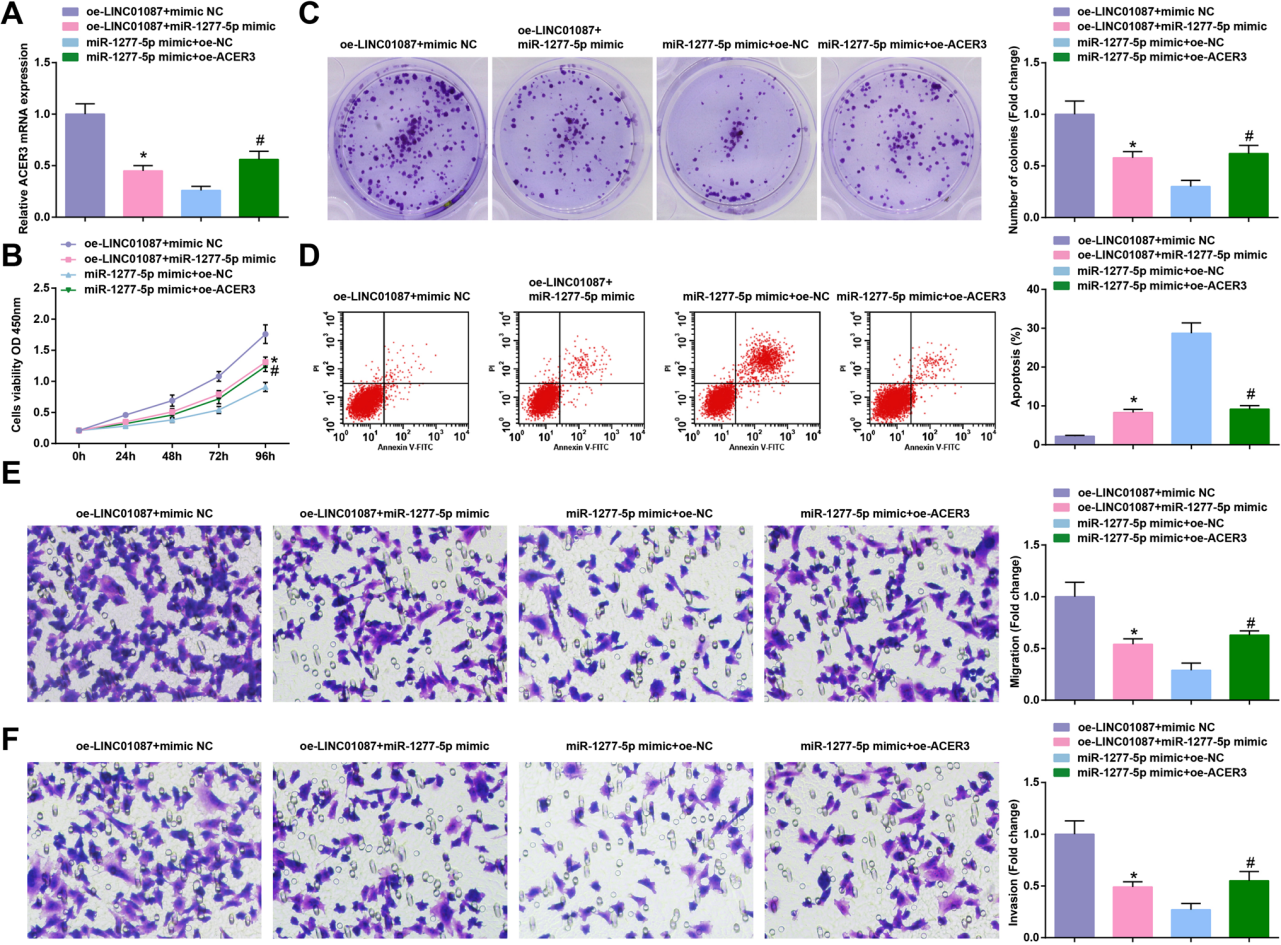
LINC01087-mediated tumorigenesis in glioma is mediated by miR-1277-5p-targeted ACER3. **A** ACER3 mRNA level assessed by RT-qPCR; **B**–**C** Cell proliferation determined by CCK-8 and colony formation assays; **D** Cell apoptosis assessed by flow cytometry; **E**–**F** Cell migration and invasion assessed by Transwell assay. The data were represented by mean ± standard deviation; *P < 0.05 vs. the oe-LINC01087 group + mimic NC group; #*P* < 0.05 vs. the miR-1277-5p mimic + oe-NC group

The integral action of LINC01087/miR-1277-5p/ACER3 axis in glioma was further evaluated. We co-transfected oe-LINC01087 + miR-1277-5p-mimic, oe-LINC01087 + mimic NC, miR-1277-5p-mimic + oe-ACER3 and miR-1277-5p-mimic + oe-N into U251 cells. Determination of cellular function highlighted that miR-1277-5p-mimic repressed the growth of U251 cells mediated by oe-LINC01087 oe-ACER3 reversed the growth inhibition of U251 cells caused by miR-1277-5p-mimic (Fig. 7B–F). In short, LINC01087-mediated pro-tumorigenesis in glioma could be reduced by overexpressing miR-1277-5p or suppressing ACER3.

## Discussion

After cerebral stroke, glioma is the second cause of death from central nervous system diseases (Bilmin et al. 2019). In this research, we evaluated the fundamental effects of LINC01087/miR-1277-5p/ACER3 axis in glioma cell growth, and attained the main observation illustrating that LINC01087 facilitated the biological behaviors of glioma cells by binding to miR-1277-5p to silence ACER3.

Based on ceRNA hypothesis, the dysregulated lncRNA-miRNA-mRNA network is constructed in triple-negative breast cancer and LINC01087 is inclusive in sub-modules (Naorem et al. 2020). LINC01087 expression was found to upregulate in patients with glioma. Based on that, we evaluated the correlation between LINC01087 expression and clinicopathological characteristics and overall survival of glioma patients, and captured the conclusion that high LINC01087 expression was associated with larger tumor size, advanced WHO stage, and dismal prognosis in glioma. Afterwards, we designed LINC01087 silencing in vitro experiments and found that decreasing LINC01087 impaired proliferation, invasion, and migration but augmented apoptosis of glioma cells. Accordingly, LINC01087 expression has been identified to upregulate in luminal breast cancer (De Palma et al. 2020). Ji-Kai She et al. have once probed that high LINC01087 expression in breast cancer indicates unfavorable survival, and loss of LINC01087 limits tumor cells to proliferate, migrate, and invade whereas stimulates apoptosis (She et al. 2020).

The present research also verified that LINC01087 could bind to miR-1277-5p to lower miR-1277-5p expression in glioma cells. Focusing on miR-1277-5p, we assessed a reduction in miR-1277-5p expression in glioma tissues and cells, and elevating miR-1277-5p levels weakened the malignant behaviors of glioma cells. Currently, Zhijian Wei et al. have evaluated that miR-1277-5p expression degrades in gastric cancer and upregulating miR-1277-5p exerts to retard tumor growth (Wei et al. 2020). Same to that, miR-1277 expression has been examined to decrease in serum samples of hepatocellular carcinoma patients, and in vitro decreasing miR-1277 could fuel cell growth but overexpressing miR-1277 works oppositely (Cao et al. 2019). Decreased miR-1277-5p expression could also be measured in Parkinson’s disease models of dopaminergic neuronblastoma SK-N-SH cells, and enhancing miR-1277-5p could exert neuroprotection against inflammation, oxidative stress, and apoptosis (Zhou et al. 2021). Besides, miR-1277-5p expression is dropped in osteoarthritis, and promotion of miR-1277-5p could function to reduce extracellular matrix degradation (Wang et al. 2019), as well as suppress chondrocyte apoptosis and inflammation (Wang et al. 2021b). Overall, artificially modifying the deregulated miR-1277-5p could attenuate the progress of diseases as suggested above.

Afterwards, ACER3, confirmed as a target of miR-1277-5p, was found to involve in glioma development. ACER3 expression was raised in glioma, and knocking down ACER3 could restrain the malignant phenotypes of gliomas while overexpressing ACER3 restrained the effects of restored miR-1277-5p on glioma cells. In fact, Ceranib-2, an inhibitor of ceramidase, is effective to utmost impede growth and drive apoptosis of human glioma cells T-98G (Kus et al. 2018). Chen Chen et al. have once mentioned that reduced ACER3 expression is related to dismal overall survival in acute myeloid leukemia, and downregulated ACER3 is essentially required to suppress cellular proliferation and augment apoptosis (Chen et al. 2016). More importantly, an article related to hepatocellular carcinoma has assessed the role of ACER3 in tumor development and revealed that ACER3 overexpression impairs radiosensitivity and apoptosis but aggrandize metastasis of HCC cells (Yang et al. 2020). Also, Yin Y et al. have reported the pro-tumor actions of ACER3 in HCC, and depression of ACER3 is of importance to block proliferative and anti-apoptotic activities of HCC cells (Yin et al. 2018). Similarly, the abnormally elevated ACER3 expression is measured in nonalcoholic steatohepatitis, and functionally inhibiting ACER3 could protect livers against inflammation and fibrosis through depressing oxidative stress (Wang et al. 2020).

All in all, our research has determined the mechanism by which LINC01087 accelerated the growth of glioma cells via downregulating miR-1277-5p and upregulating ACER3. This study design has innovatively observed cellular progression in glioma with the interference with LINC01087/miR-1277-5p/ACER3 and renewed a theoretical basis for searching for novel agent to manage glioma development. However, much more efforts are still required to explore the downstream signaling pathways related to the progression of glioma mediated by the LINC01087/miR-1277-5p/ACER3 axis.

## References

[CR1] Bilmin K, Kujawska T, Grieb P (2019) Sonodynamic therapy for gliomas. Perspectives and prospects of selective sonosensitization of glioma cells. Cells. 8(11) 10.3390/cells8111428 PMC6912826 31766152

[CR2] Bush NA, Chang SM, Berger MS (2017). Current and future strategies for treatment of glioma. Neurosurg Rev.

[CR3] Cao X (2019). MicroRNA-1277 inhibits proliferation and migration of hepatocellular carcinoma HepG2 cells by targeting and suppressing BMP4 expression and reflects the significant indicative role in hepatocellular carcinoma pathology and diagnosis after magnetic resonance imaging assessment. Oncol Res.

[CR4] Chen C (2016). ACER3 supports development of acute myeloid leukemia. Biochem Biophys Res Commun.

[CR5] Coant N (2017). Ceramidases, roles in sphingolipid metabolism and in health and disease. Adv Biol Regul.

[CR6] Dang Y (2018). Long non-coding RNA in glioma: target miRNA and signaling pathways. Clin Lab.

[CR7] Davis ME (2018). Epidemiology and overview of gliomas. Semin Oncol Nurs.

[CR8] De Palma FDE (2020). The abundance of the long intergenic non-coding RNA 01087 differentiates between luminal and triple-negative breast cancers and predicts patient outcome. Pharmacol Res.

[CR9] Gusyatiner O, Hegi ME (2018). Glioma epigenetics: from subclassification to novel treatment options. Semin Cancer Biol.

[CR10] Kus G (2018). Comparison of a ceramidase inhibitor (ceranib-2) with C2 ceramide and cisplatin on cytotoxicity and apoptosis of glioma cells. Turk J Biol.

[CR11] Li Z (2020). lncRNA CRNDE promotes the proliferation and metastasis by acting as sponge miR-539-5p to regulate POU2F1 expression in HCC. BMC Cancer.

[CR12] Liu X (2019). LncRNA LINC00689 promotes the growth, metastasis and glycolysis of glioma cells by targeting miR-338–3p/PKM2 axis. Biomed Pharmacother.

[CR13] Liu C et al (2020) LncRNA XIST promotes the progression of laryngeal squamous cell carcinoma via sponging miR-125b-5p to modulate TRIB2. Biosci Rep. 40(4) 10.1042/BSR20193172 PMC7146034 32149330

[CR14] Naorem LD (2020). Comprehensive analysis of dysregulated lncRNAs and their competing endogenous RNA network in triple-negative breast cancer. Int J Biol Macromol.

[CR15] Ostrom QT (2014). The epidemiology of glioma in adults: a “state of the science” review. Neuro Oncol.

[CR16] She JK (2020). LINC01087 is highly expressed in breast cancer and regulates the malignant behavior of cancer cells through miR-335-5p/Rock1. Onco Targets Ther.

[CR17] Shi J (2019). Long non-coding RNA LINC00174 promotes glycolysis and tumor progression by regulating miR-152-3p/SLC2A1 axis in glioma. J Exp Clin Cancer Res.

[CR18] Tom MC (2019). Management for different glioma subtypes: are all low-grade gliomas created equal?. Am Soc Clin Oncol Educ Book.

[CR19] Tripathi R (2020). Unravelling the role of long non-coding RNA - LINC01087 in breast cancer. Noncoding RNA Res.

[CR20] Wang T (2019). Long non-coding RNA XIST promotes extracellular matrix degradation by functioning as a competing endogenous RNA of miR-1277-5p in osteoarthritis. Int J Mol Med.

[CR21] Wang K (2020). Targeting alkaline ceramidase 3 alleviates the severity of nonalcoholic steatohepatitis by reducing oxidative stress. Cell Death Dis.

[CR22] Wang Z (2021). Rapamycin inhibits glioma cells growth and promotes autophagy by miR-26a-5p/DAPK1 axis. Cancer Manag Res.

[CR23] Wang B (2021). LncRNA HOTAIR modulates chondrocyte apoptosis and inflammation in osteoarthritis via regulating miR-1277-5p/SGTB axis. Wound Repair Regen.

[CR24] Wei Z (2020). LncRNA HOTAIR promotes the growth and metastasis of gastric cancer by sponging miR-1277-5p and upregulating COL5A1. Gastric Cancer.

[CR25] Yang G (2020). LncRNA KCNQ1OT1 inhibits the radiosensitivity and promotes the tumorigenesis of hepatocellular carcinoma via the miR-146a-5p/ACER3 axis. Cell Cycle.

[CR26] Yin Y (2018). Alkaline ceramidase 3 promotes growth of hepatocellular carcinoma cells via regulating S1P/S1PR2/PI3K/AKT signaling. Pathol Res Pract.

[CR27] Zhang W (2020). Circ-ELF2 acts as a competing endogenous RNA to facilitate glioma cell proliferation and aggressiveness by targeting MiR-510-5p/MUC15 signaling. Onco Targets Ther.

[CR28] Zhou S (2021). Deficiency of NEAT1 prevented MPP(+)-induced inflammatory response, oxidative stress and apoptosis in dopaminergic SK-N-SH neuroblastoma cells via miR-1277–5p/ARHGAP26 axis. Brain Res.

